# Activity of tumor-associated macrophage depletion by CSF1R blockade is highly dependent on the tumor model and timing of treatment

**DOI:** 10.1007/s00262-021-02861-3

**Published:** 2021-01-29

**Authors:** Sarah A. O’Brien, Jessica Orf, Katarzyna M. Skrzypczynska, Hong Tan, Jennie Kim, Jason DeVoss, Brian Belmontes, Jackson G. Egen

**Affiliations:** Department of Inflammation and Oncology, Amgen Research, Amgen Inc., South San Francisco, CA 94080 USA

**Keywords:** Cancer immunotherapy, Immunosuppression, T cell, Tumor microenvironment, TAM

## Abstract

Tumor-associated macrophages (TAMs) are abundant in solid tumors where they exhibit immunosuppressive and pro-tumorigenic functions. Inhibition of TAM proliferation and survival through CSF1R blockade has been widely explored as a cancer immunotherapy. To further define mechanisms regulating CSF1R-targeted therapies, we systematically evaluated the effect of anti-CSF1R treatment on tumor growth and tumor microenvironment (TME) inflammation across multiple murine models. Despite substantial macrophage depletion, anti-CSF1R had minimal effects on the anti-tumor immune response in mice bearing established tumors. In contrast, anti-CSF1R treatment concurrent with tumor implantation resulted in more robust tumor growth inhibition and evidence of enhanced anti-tumor immunity. Our findings suggest only minor contributions of CSF1R-dependent TAMs to the inflammatory state of the TME in established tumors, that immune landscape heterogeneity across different tumor models can influence anti-CSF1R activity, and that alternative treatment schedules and/or TAM depletion strategies may be needed to maximize the clinical benefit of this approach.

**Supplementary information:**

The online version of this article (10.1007/s00262-021-02861-3) contains supplementary material, which is available to authorized users.

## Introduction

Tumor-associated macrophages (TAMs) are an abundant and highly heterogeneous immune cell population within solid tumors that are generally thought to have pro-tumorigenic functions [1]. Colony stimulating factor 1 receptor (CSF1R) is a receptor tyrosine kinase that promotes survival, proliferation, and differentiation of monocytes and macrophages downstream of interactions with CSF1 and IL34 in normal tissues and tumors. As such, inhibition of CSF1R signaling using antagonist antibodies or small molecule inhibitors has been widely studied as an immunotherapy for solid tumors in both mouse and human [2]. In some mouse tumor models, blockade of CSF1R has been shown to dramatically reduce TAM density [3, 4], or promote the induction of pro-inflammatory TAM phenotypes [5, 6], leading to immune activation and tumor regression [7]. However, the reported effects of CSF1R inhibitor therapy on the inflammatory state of the tumor microenvironment (TME) and tumor growth vary widely [1, 3, 8–14]. Likewise, in the clinic, CSF1R inhibition has led to robust macrophage depletion in both normal tissues and solid tumors; however, minimal anti-tumor efficacy has been observed [4, 15, 16]. Emerging studies in mice have suggested multiple mechanisms of resistance to CSF1R inhibition, including compensatory activation of regulatory T cells (Tregs), recruitment of other suppressive myeloid populations, and resistance of pro-tumorigenic macrophage subsets to treatment [17–20], which may at least partially explain these clinical observations.

To further understand the mechanisms regulating the anti-tumor activity of CSF1R inhibition, we systematically evaluated the impact of an anti-mouse CSF1R blocking antibody on tumor growth and the TME phenotype across multiple syngeneic mouse tumor models. Despite significant depletion of TAMs in established tumors, minimal effects were observed on tumor growth and TME inflammation across most tumor models. In contrast, enhanced tumor growth inhibition was observed when anti-CSF1R treatment was initiated early after tumor implantation, which was accompanied by an increase in TME inflammation. These data provide insight into the temporal role of TAMs and myeloid-targeted therapies in regulating the inflammatory state of the TME.

## Materials and methods

### Mice, cell lines, and tumor studies

All mice were used in accordance with the National Institutes of Health guidelines and experiments were approved by the Amgen Institutional Animal Care and Use Committee. Female 6–8-week-old BALB/c or C57BL/6 mice were from Charles River Laboratories. 3 × 10^5^ CT26, RENCA, EMT6, LL2, and MC38 and 2 × 10^5^ B16F10 cells were subcutaneously injected into the right flank. Animal weights and tumor volumes (LxWxH) were measured twice weekly throughout the study. Starting on the day of tumor implantation (day 0), or when mean tumor volume were approximately 100 mm^3^ (day 10–14), 400 µg/mouse a murine IgG1 isotype control antibody (BioXCell; clone MOPC-21) or anti-CSF1R antibody [21] were injected intraperitoneally (i.p.) 3 times weekly. Isotype control muIgG2a antibody (BioXCell; clone 2A3) or anti-CD8α (BioXCell; clone 53–6.7) were dosed i.p. twice weekly; initial dose of 500 µg/mouse and subsequent doses of 200 µg/mouse.

### Flow cytometry

Single-cell suspensions were prepared from tumors using the GentleMacs Octo instrument (Miltenyi) and digested enzymatically in media containing Liberase TL (Roche, 0.2 mg/ml) and DNase I (Roche, 20 U/ml). Surface marker staining was performed in the presence of purified CD16/CD32 antibody (BD Biosciences) using the following antibodies: Ly6C (HK1.4), Ly6G (1A8), F4/80 (BM8), MHCII (M5/114.15.2), PD-L1 (10F.9G2), CD206 (C068C2), Thy1.2 (30-H12), CD4 (GK1.5), CD8 (53–6.7, Biolegend), CD45 (30-F11), CD11b (M1/70), ICOS (7E.17G), and TCRβ (H57-597, BD Bioscience). CountBright Counting Beads (Invitrogen) were added to fixed volumes of single-cell suspensions and flow cytometry was used to determine absolute cell numbers of different immune cell populations, according to the manufacturer’s instructions. Fixable Aqua Dead Cell Staining kit was used to discriminate live cells (Invitrogen). For intracellular cytokine staining, cells were incubated for 3 h at 37 °C with cell stimulation cocktail plus protein transport inhibitors or protein transport inhibitor only (ThermoFisher). Cells were fixed/permeabilized using Foxp3/Transcription Factor staining kit (eBioscience) and intracellular stained with Foxp3 (FJK-16 s), Ki67 (SolA15), IL12p40 (C17.8), IL6 (MP5-20F3), or IFNγ (XMG1.2, eBioscience). Data were acquired on the LSRII or FACSymphony (BD Biosciences) and analyzed using FlowJo software (Treestar).

### Real-time PCR

Total RNAs were purified from snap frozen tumors using the GentleMacs Octo instrument (Miltenyi) and the RNeasy Plus Kit (Qiagen). cDNAs were synthesized with the High Capacity cDNA Reverse Transcription Kit (Applied Biosystems) and preamplification were performed using TaqMan PreAmp Master Mix (Applied Biosystems). High-throughput quantitative real-time PCR (qRT-PCR) for 96 genes was done using primer–probe sets from Integrated DNA Technologies on a 96.96 dynamic array (Fluidigm). Target gene expression was normalized to the mean of *Ipo8*, *Tbp*, and *Hrpt* housekeeping genes using the dCt method. Data analysis was performed using Spotfire (Tibco) and Array Studio software (Omicsoft Corporation).

### Statistics

Data were graphed and analyzed in GraphPad Prism. Tumor growth is shown as mean tumor volume ± SEM over time for each treatment group. Area under the tumor growth curve was calculated for each animal using the midpoint rule approximation and statistical analysis performed on these data using unpaired, two-tailed t-test or one-way ANOVA with Tukey’s (all possible comparisons) or Sidak’s (select comparisons) multiple testing correction. Flow cytometry data are shown as mean + SD and statistics were analyzed using unpaired, two-tailed t test or one-way ANOVA with Tukey’s multiple comparisons.

## Results and discussion

### Minimal effect of anti-CSF1R treatment on the growth or inflammatory state of established syngeneic tumors

**Fig. 1:**
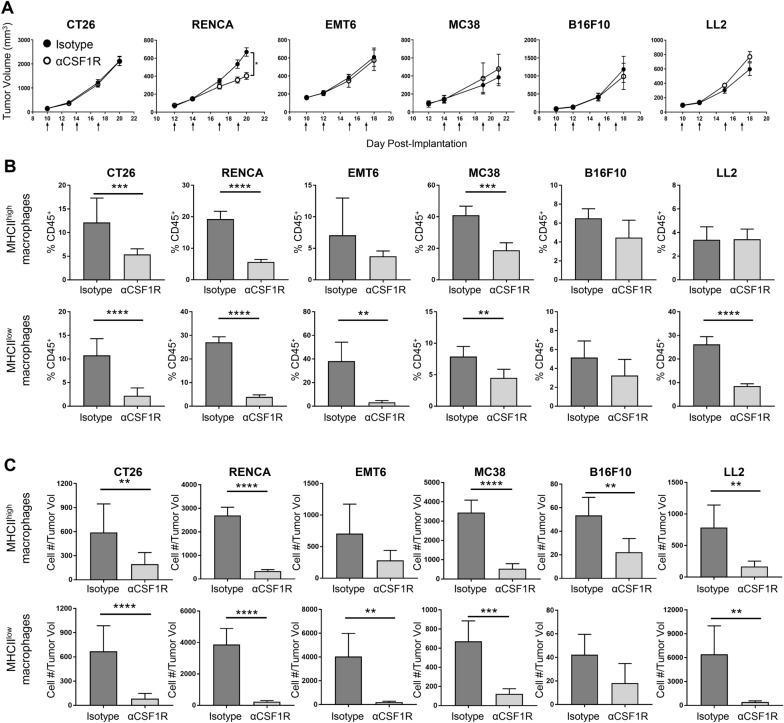
αCSF1R treatment has minimal effects on tumor growth despite significant TAM depletion. **a** Growth curves for the indicated tumor models from mice treated with αCSF1R or isotype control antibody as indicated by arrows; *n* = 5–12 animals/group. Two-tailed *t* test. **b**, **c** F4/80^+^MHCII^high^ and F4/80^+^MHCII^low^ TAM populations identified by flow cytometry on tumors harvested at the end of study are shown as percentage of CD45^+^ immune cells (**b**) or number of cells normalized to individual take-down tumor volume (**c**); *n* = 5–10 animals/group. Two-tailed *t* test. *p < 0.05; **p < 0.01; ***p < 0.001; ****p < 0.0001

To evaluate the effects of anti-CSF1R blockade on solid tumors, we treated BALB/c mice bearing established CT26, RENCA, or EMT6 tumors and C57BL/6 mice bearing established MC38, B16F10, or LL2 tumors with an anti-mouse CSF1R (αCSF1R) antagonist antibody [21], monitoring tumor growth over time and characterizing the phenotype of tumor-associated immune cells at the end of study using flow cytometry. Interestingly, most models showed no effect of αCSF1R treatment on tumor growth, except for the RENCA model, where a modest but significant decrease in tumor volume was observed (Fig. 1a).

We next characterized the effect of αCSF1R treatment on TAM populations across the different tumor models, quantifying F4/80^+^ MHC class II (MHCII)^high^ and MHCII^low^ TAM subsets by flow cytometry (Supplemental Fig. 1a, 1b). This gating strategy was based on previous publications suggesting anti- and pro-tumorigenic properties associated with these phenotypes [22, 23]. Assessment of both macrophage frequency in the tumor as a percentage of all immune cells (Fig. 1b) and macrophage density (Fig. 1c), defined as the total number of TAMs/tumor volume, demonstrated significant αCSF1R-mediated TAM depletion in all models, with variability in magnitude across the MHCII^high^ and MHCII^low^ populations. For instance, in B16F10 tumors, which contain a relatively low frequency of both TAM subsets, only moderate effects of αCSF1R treatment on TAM density were observed, only reaching statistical significance for the MHCII^high^ population (Fig. 1c). These data reveal highly heterogeneous effects of αCSF1R treatment on TAM populations across various tumor models and suggest that tumors with higher baseline TAM density show more robust αCSF1R-mediated TAM depletion, likely due to a greater degree of CSF1-dependent macrophage expansion and preferential sensitivity of proliferating TAMs to CSF1R inhibition [20]. Notably, the efficient αCSF1R-mediated TAM depletion observed across the multiple tumor models evaluated did not universally translate to effects on tumor growth.

We also examined the effect of αCSF1R treatment on other myeloid cell populations, including neutrophils, Ly6C^high^ classical monocytes, and dendritic cells (Supplemental Fig. 2). In RENCA, EMT6, and LL2 models, αCSF1R treatment was found to induce an increase in the frequency of tumor-associated neutrophils, yet only RENCA and EMT6 αCSF1R-treated tumors had a significant increase in neutrophil cell density. The increase in neutrophil recruitment after αCSF1R treatment is consistent with previous studies demonstrating CSF1R inhibition promotes cancer-associated fibroblasts to secrete chemokines involved in neutrophil recruitment [18]. However, notably, an αCSF1R-mediated increase in neutrophils was not observed across all tumor models, potentially relating to differences in fibroblast content or phenotype.

**Fig. 2:**
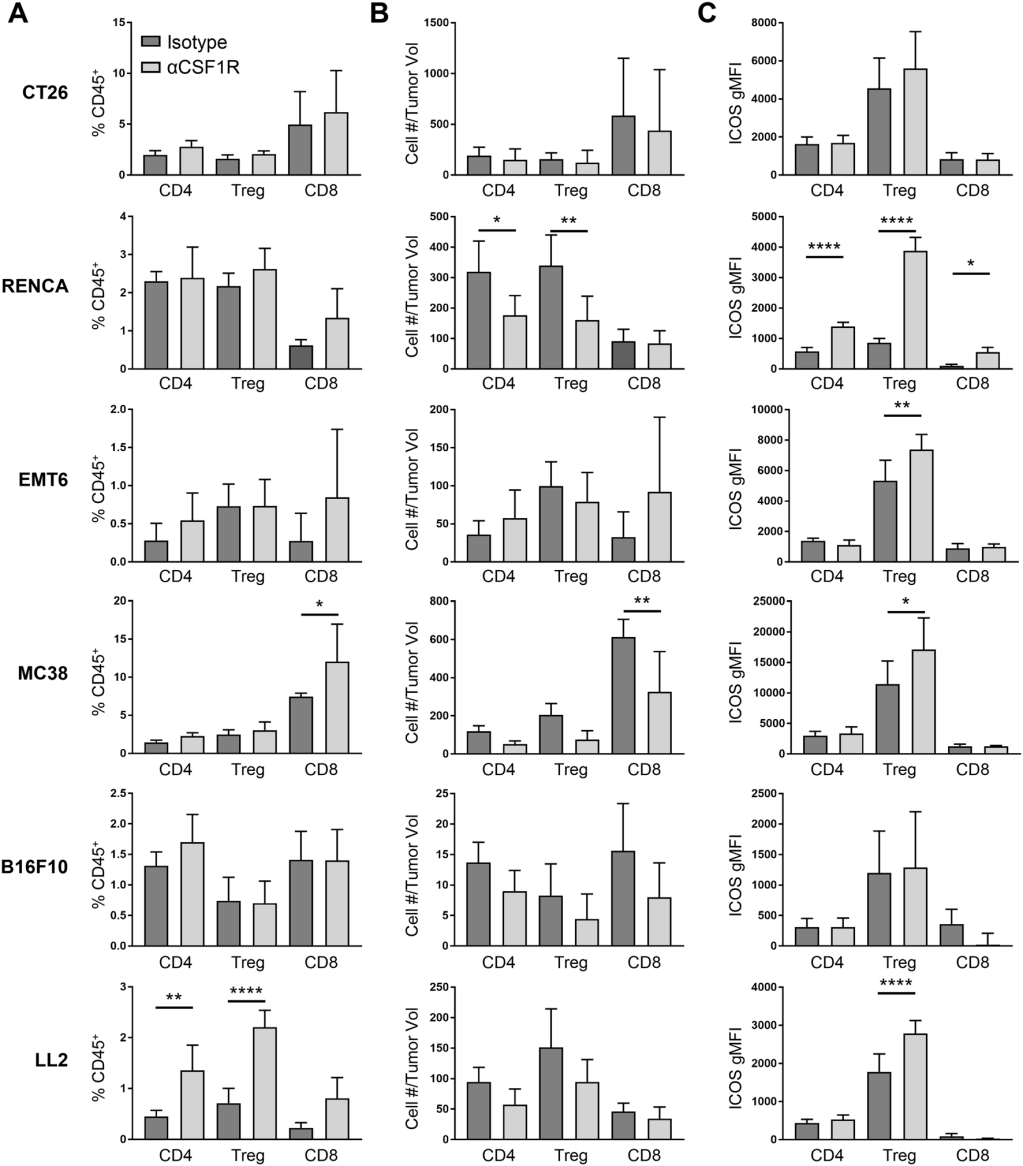
αCSF1R treatment has varying effects on T cell density and activation across different tumor models. **a**–**c** Flow cytometry analysis of tumors from αCSF1R- or isotype control-treated animals. T cells were subdivided into CD4^+^Foxp3^−^ (CD4), CD4^+^Foxp3^+^ (Treg), or CD8^+^ (CD8) subsets and are shown as percentage of CD45^+^ immune cells (**a**) or number of cells normalized to individual tumor volume (mm^3^) at the end of study (**b**). **c** Geometric mean fluorescence intensity (gMFI) for ICOS staining on different T cell populations; *n* = 5–10 animals/group. One-way ANOVA, Tukey’s multiple comparisons. *p < 0.05; **p < 0.01; ****p < 0.0001

CSF1 blockade has also been reported to alter the TME, leading to increased T cell recruitment and activation [9–11, 14]. To further understand the effect of TAM depletion on the inflammatory state of the TME, we quantified tumor-associated CD8^+^ T cells, CD4^+^ T regulatory cells (Tregs), and CD4^+^ non-Tregs across the various tumor models (Supplemental Fig. 1c). Following αCSF1R treatment, CT26, EMT6, and B16F10 tumors showed no significant change in either T cell frequency as a percentage of CD45^+^ cells or T cell density (Fig. 2a, b). In contrast, MC38 tumors had a significant increase in the percentage of tumor-associated CD8^+^ T cells after αCSF1R treatment, likely due to a decrease in TAMs from the total CD45^+^ population without a compensatory increase in another abundant myeloid population (Supplemental Fig. 2). Indeed, the overall density of CD8^+^ T cells was reduced compared to control-treated animals in this model, corresponding to marginally increased tumor volumes following αCSF1R treatment (Fig. 1a). A similar observation was made in the LL2 model for CD4^+^ T cell populations. Interestingly, following αCSF1R treatment of mice bearing RENCA tumors, a reduction in the density of CD4^+^ Tregs and non-Tregs, but not CD8^+^ T cells, was observed (Fig. 2a, b). A heatmap summary of the αCSF1R-mediated effects on immune cell density across all populations examined in our study is provided in Supplemental Fig. 2E.

Finally, the effect of αCSF1R treatment on T cell activation status was examined, focusing on the surface marker ICOS based on preliminary data showing induction of ICOS gene expression in whole tumor lysates and previous studies associating ICOS upregulation on tumor T cells with enhanced anti-tumor immune responses following immunotherapy [24–27]. ICOS signaling can promote proliferation and cytokine production by conventional T cells but may also promote the immunosuppressive activity of Tregs [28]. Interestingly, we found that CD4^+^ Tregs upregulated ICOS in multiple tumor models following αCSF1R treatment (Fig. 2c). While the effect of this change in phenotype on Treg function is unknown, these data are suggestive of TAM-Treg crosstalk and consistent with previous demonstrations of compensatory enhancement of Treg-mediated immunosuppression in tumors following TAM depletion [17]. Together, these data suggest that despite robust depletion of TAMs, αCSF1R therapy fails to promote anti-tumor activity of the T cell compartment and may instead potentiate Treg-mediated immunosuppression. Notably, only RENCA tumors had significantly upregulated ICOS expression in CD4^+^ non-Tregs and CD8^+^ T cells after αCSF1R treatment (Fig. 2c), which along with the effects on CD8^+^ T cell and Treg densities described above, may explain the αCSF1R-mediated tumor growth inhibition observed in this model.

### Timing of anti-CSF1R treatment is a determinant of its ability to inhibit tumor growth and potentiate anti-tumor T cell responses

**Fig. 3:**
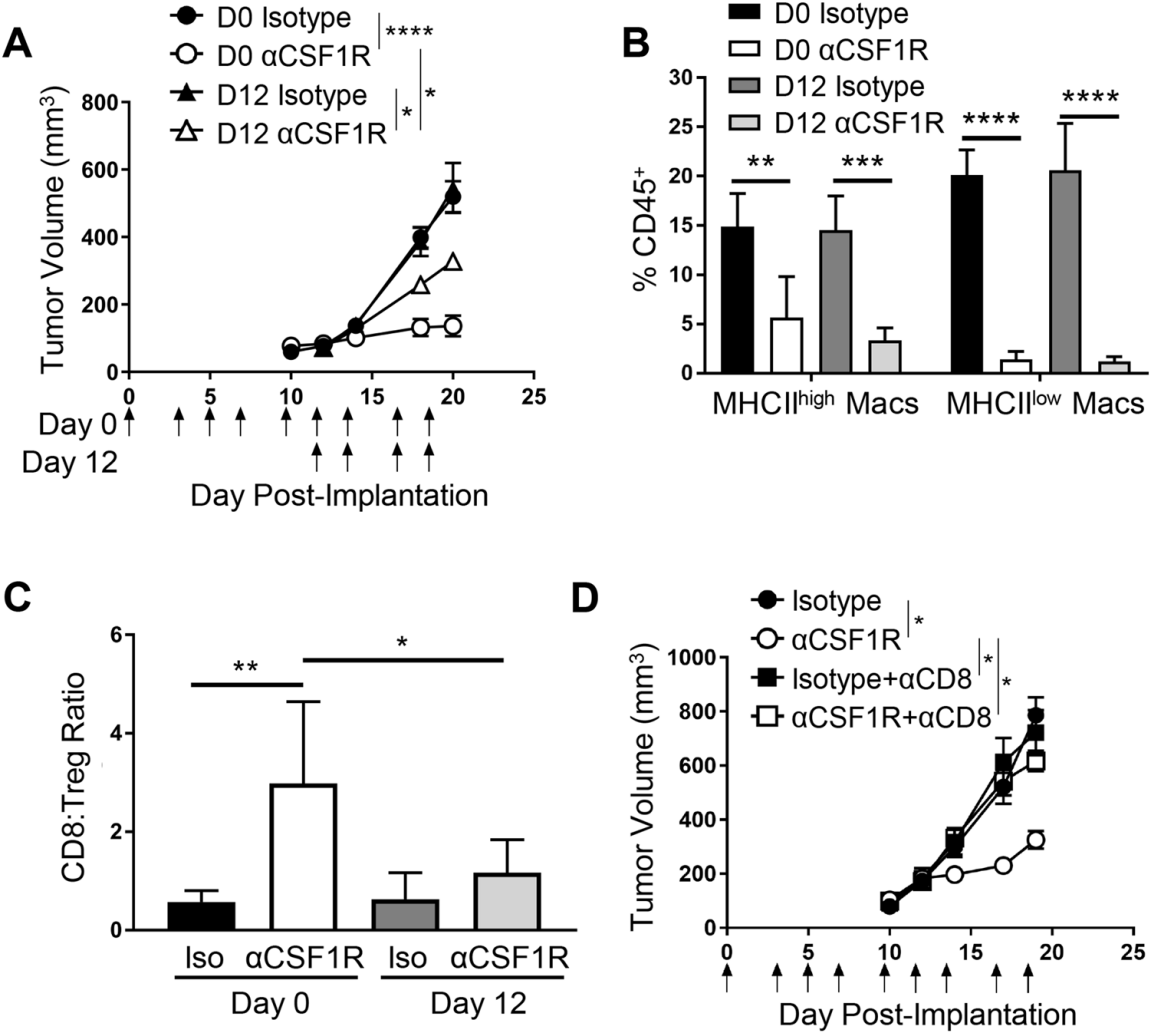
Early initiation of αCSF1R treatment enhances anti-tumor efficacy through CD8^+^ T cell-mediated immune responses. **a**–**c** Early vs late treatment with αCSF1R in RENCA model. **a** Tumor growth curves for mice treated as indicated by arrows starting on day 0 or 12 post-tumor implantation; *n* = 9–10 animals/group. One-way ANOVA, Sidak’s multiple comparisons (αCSF1R vs isotype control at D0 and D12, αCSF1R D0 vs αCSF1R D12). **b**, **c** TAM depletion (**b**) and CD8^+^ T cell-to-Treg ratios (**c**) in tumor as measured by flow cytometry; *n* = 5 animals/group. **d** Tumor growth curves for RENCA tumor-bearing mice treated at day 0 with αCSF1R, αCD8, or control antibodies, as indicated; *n* = 10 animals/group. One-way ANOVA, Tukey’s multiple comparisons. *p < 0.05; **p < 0.01; ***p < 0.001; ****p < 0.0001

Given that treatment of established RENCA tumors with αCSF1R led to a modest but reproducible decrease in tumor growth that was accompanied by a significant effect on T cell abundance and phenotype, we next examined the relationship between treatment timing and response in this model. Mice were dosed with αCSF1R or control antibody starting on the day of tumor implantation (day 0) or when tumors were ~ 100mm^3^ (day 12) and continually treated 3 times per week until day 20. Interestingly, we observed that initiating treatment at day 0 resulted in greater tumor growth inhibition compared to day 12 (Fig. 3a). Examining tumors by flow cytometry at the end of study revealed that the extent of αCSF1R-mediated TAM depletion was similar between the two dosing groups (Fig. 3b), suggesting that prolonged duration of αCSF1R exposure with early treatment does not result in greater TAM depletion or contribute to the observed differences in tumor growth. αCSF1R-mediated neutrophil recruitment was also similar with early and late treatment (Supplemental Fig. 3a), suggesting that neutrophil influx is not responsible for the observed differences in efficacy with these two treatment regimens.

The observed increase in anti-tumor efficacy resulting from early αCSF1R dosing could be due to the role of macrophages in promoting the initial survival and growth of tumor cells following transplantation, establishment of tumor vascularization through effects on angiogenesis, or a more pronounced role for TAMs in regulating the inflammatory state of the TME during tumor formation [29]. To investigate this latter possibility, we examined the effect of early versus late αCSF1R treatment on tumor T cell populations. Interestingly, we observed a preferential increase in the CD8^+^ T cell-to-Treg ratio in RENCA tumors following initiation of αCSF1R treatment on day 0 compared to day 12 (Fig. 3c), driven by an increase in the frequency of CD8^+^ T cells rather than a change in the proportions of Tregs (Supplemental Fig. 3b). αCSF1R-mediated induction of ICOS on Tregs was also similar between treatment regimens (Supplemental Fig. 3c), suggesting that differential effects on the Treg compartment are not leading to the observed differences in tumor growth with early vs late αCSF1R treatment.

To determine the role of CD8^+^ T cells in tumor growth inhibition observed with early αCSF1R treatment, mice were treated with an anti-CD8 (αCD8) depleting antibody concurrent with early initiation of αCSF1R therapy. Consistent with the previous results, macrophage depletion starting at day 0 resulted in robust tumor growth inhibition and this effect was completely abrogated in CD8^+^ T cell-depleted animals (Fig. 3d). Flow cytometry on disassociated tumors confirmed that αCD8 treatment did not impact αCSF1R-mediated TAM depletion (Supplemental Fig. 3d) and that αCSF1R treatment did not impact αCD8-mediated T cell depletion (Supplemental Fig. 3e). Taken together, these data indicate that the effects of early TAM depletion on RENCA tumor growth are dependent on potentiation of an adaptive immune response against the tumor, consistent with previous publications demonstrating that TAMs can inhibit CD8^+^ T cell responses [9, 14]. The dramatic difference in tumor growth inhibition observed between early and late αCSF1R treatment, despite similar TAM depletion, suggests that macrophage depletion from developing tumors has a greater ability to promote TME inflammation and potentiate anti-tumor CD8^+^ T cell responses.

### Early, but not late, anti-CSF1R treatment drives robust inflammatory responses in tumor

**Fig. 4:**
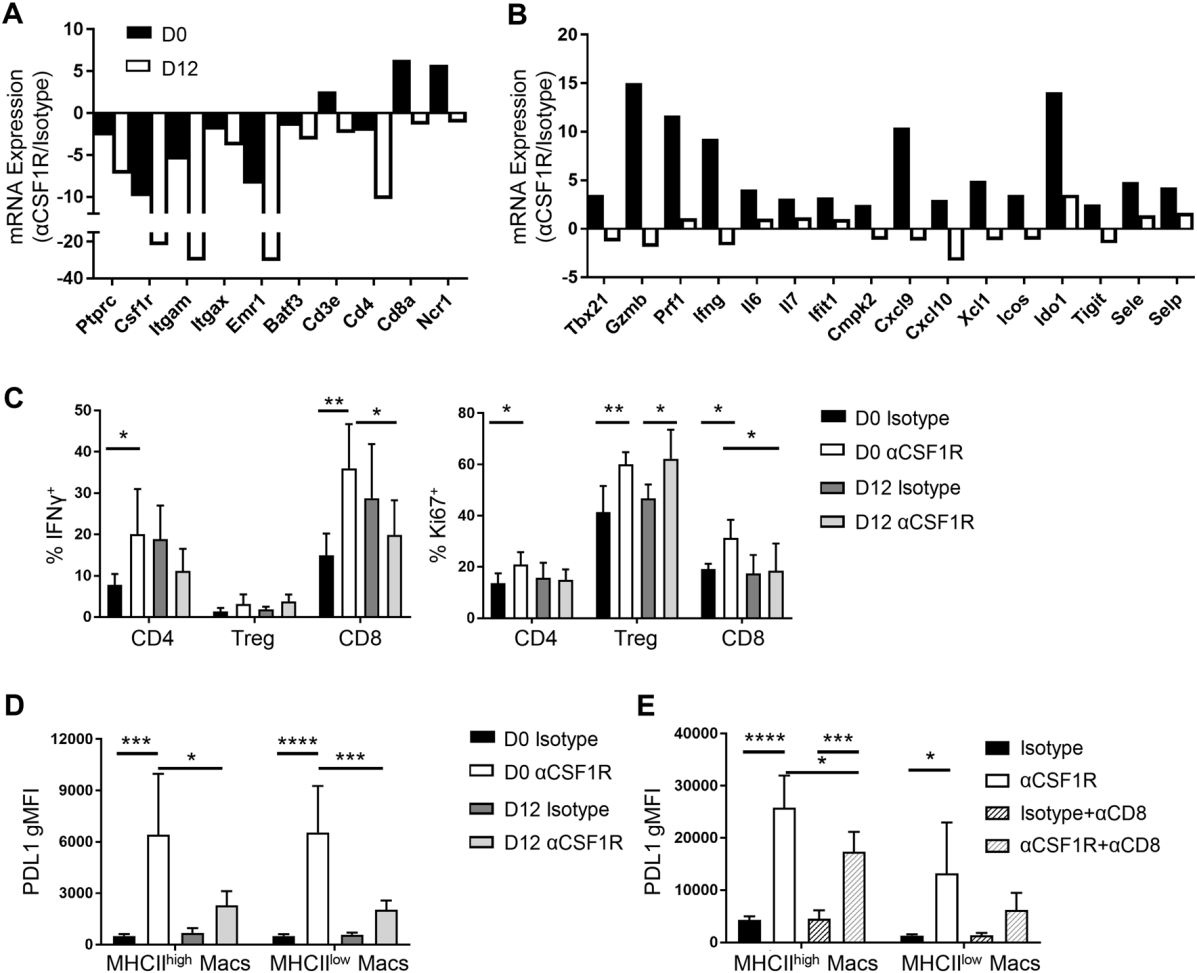
Early dosing of αCSF1R drives robust potentiation of anti-tumor immunity. **a**–**d** RENCA tumors isolated from mice treated at day 0 or 12 with αCSF1R or control antibodies. **a**, **b** Fluidigm qRT-PCR analysis. Graphs represent average—dCT values for αCSF1R over isotype-treated animals; *n* = 4–5 animals/group. **c**, **d** Frequency of IFNγ or Ki67 expressing tumor-associated T cells (**c**) and gMFI of PD-L1 staining on TAMs (**d**); *n* = 5–7 animals/group. **e** gMFI of PD-L1 staining on TAMs isolated from RENCA tumors treated with αCSF1R and αCD8 as in Fig. 3d; *n* = 5 animals/group. One-way ANOVA, Tukey’s multiple comparisons. *p < 0.05; **p < 0.01, ***p < 0.001, ****p < 0.0001

To gain insight into the mechanisms underlying potentiation of CD8^+^ T cell-mediated tumor regression following early αCSF1R treatment in RENCA tumors, we further characterized the anti-tumor immune response following early and late αCSF1R treatment. Whole tumor gene expression analysis revealed an expected αCSF1R-mediated decrease in myeloid lineage genes, including *Csf1r, Itgam, Itgax, Emr1* (F4/80), regardless of when treatment was initiated (Fig. 4a and Supplemental Fig. 4), consistent with observations using flow cytometry (Fig. 3b). Expression of genes encoding lymphocyte lineage markers was found to be increased with early, but not late, αCSF1R treatment (Fig. 4a). Interestingly, we observed that genes associated with CD8^+^ T cell recruitment and effector response, such as *Cxcl9*, *Cxcl10, Tbx21* (T-bet), *Gzmb*, *Prf1*, and *Ifng*, were uniquely upregulated following early αCSF1R treatment, along with other pro-inflammatory factors, immune activation markers, and interferon (IFN)-response genes (Fig. 4b). These data suggest that early TAM depletion can potentiate TME inflammation and drive enhanced anti-tumor cytolytic T cell responses.

We next conducted flow cytometry analysis of RENCA tumors to relate αCSF1R-induced changes in pro-inflammatory gene expression to changes in specific immune cell phenotypes. Analysis of T cell populations revealed a greater percentage of IFNγ-expressing CD4^+^ non-Tregs and CD8^+^ T cells in day 0 αCSF1R-treated mice relative to control-treated mice that was not seen in day 12 treated animals (Fig. 4c). Expression of the proliferation marker Ki67 was also preferentially increased in all T cell populations following early αCSF1R treatment, while late αCSF1R treatment only increased Ki67 expression in Tregs (Fig. 4c). We also evaluated the TAM phenotype, finding higher expression of PDL1 on both MHCII^high^ and MHCII^low^ macrophages with early compared to late αCSF1R treatment (Fig. 4d). A potential explanation for these findings is the preferential depletion of PDL1^low^ TAMs with early αCSF1R treatment, leaving PDL1^high^ subsets remaining in the tumor. Consistent with this hypothesis, we had previously found that αCSF1R-resistant macrophages in the RENCA model express higher levels of PDL1 [20]. Alternatively, PDL1 induction on the remaining macrophages following early αCSF1R treatment could reflect a macrophage response to changes in the TME. Given that early αCSF1R treatment led to a higher frequency of IFNγ-expressing T cells and IFNγ is known to induce myeloid PD-L1 expression [30], we next wanted to understand whether IFNγ production by T cell can contribute to the upregulation of PD-L1 expression. To this end, we compared PD-L1 expression by TAMs in mice treated with both early αCSF1R and an αCD8 depleting antibody, finding that PD-L1 expression was reduced in the absence of CD8^+^ T cells compared to treatment with αCSF1R alone (Fig. 4e), suggestive of cross-talk between the CD8^+^ T cell and TAM compartments.

Finally, we compared the effect of early and late αCSF1R treatment in the CT26 model, observing a similar, albeit less dramatic, effect of early αCSF1R treatment on tumor growth as in the RENCA model (Supplemental Fig. 5a), despite a similar extent of TAM depletion between the two dosing regimens (Supplemental Fig. 5b). Notably, in contrast to the RENCA model, we did not observe changes in CD8^+^ T cell or Treg frequency (Supplemental Fig. 5c) or the CD8^+^ T cell-to-Treg ratio (Supplemental Fig. 5d) with early αCSF1R treatment. However, whole tumor gene expression analysis did reveal a modest upregulation of NK cell markers and IFNγ cytokine expression with early, compared to late, αCSF1R treatment (Supplemental Fig. 5e), which may be indicative of an enhanced anti-tumor immune response, and related to the modest tumor growth inhibition with this dosing regimen. The differing responses of αCSF1R treatment observed between CT26 and RENCA highlights the different consequences of TAM depletion across mouse tumor models. Future studies aimed at characterizing TAM subset heterogeneity, spatial localization within tumors, and interaction with other immune and stroma populations may elucidate mechanisms responsible for the variable effects of αCSF1R treatment observed with different tumor models and treatment regimens.

Our data suggest that early and sustained TAM depletion during tumor formation can induce robust TME inflammation associated with immune-mediated tumor growth inhibition. While the mechanisms responsible for the differences between early and late αCSF1R treatment are not known, we speculate that early loss of macrophage-mediated clearance of dying tumor cells during tumor engraftment and initiation may lead to accumulation of immunostimulatory ligands, such as danger-associated molecular patterns (DAMPs), which can promote type I IFN production. This hypothesis is consistent with previous findings [31] and with our observation that PD-L1 and other IFN-response genes are preferentially induced with early αCSF1R treatment. Importantly, type I IFN plays a critical role in the induction of anti-tumor immunity by potentiating the ability of cross-presenting dendritic cells to initiate tumor-specific CD8^+^ T cell responses [32, 33]. Depletion of TAMs may also reduce levels of immunosuppressive cytokines, such as IL10 and TGFβ, which can inhibit the function of DCs and T cells [1], or directly promote interactions between tumor-associated T cells and low abundant cross-presenting DCs in the TME [34]. Potentiation of tumor-specific T cells through these mechanisms at early time points following tumor initiation may provide time for the immune response to develop and subsequently control the growth of rapidly proliferating tumor cells. Finally, we have previously demonstrated that established murine tumors contain heterogeneous subsets of TAMs with differential sensitivity to αCSF1R [20]. As TAMs with a pro-tumorigenic phenotype were resistant to αCSF1R-mediated depletion, it is possible that early versus late αCSF1R treatment could differentially affect the distribution of pro- and anti-tumorigenic TAM populations. Taken together, our findings suggest that TAMs have distinct and varied roles during the process of tumor formation, and the timing and duration of CSF1R inhibitor treatment may be a critical factor in determining the activity of this therapeutic approach.

## Supplementary information

Below is the link to the supplementary information.

Supplementary material 1 (PDF 943 kb)

## Data Availability

All data generated or analyzed during this study are included in this published article and its supplementary files.
