# Supplementary material for: Activity of tumor-associated macrophage depletion by CSF1R blockade is highly dependent on the tumor model and timing of treatment
Source: Cancer Immunol Immunother. 2021 Jan 29;70(8):2401–10. doi: 10.1007/s00262-021-02861-3 (PMC8289806; doi:10.1007/s00262-021-02861-3)
Supplement: Supplementary file 1 — Supplementary material 1 (PDF 943 kb) [file 262_2021_2861_MOESM1_ESM.pdf]

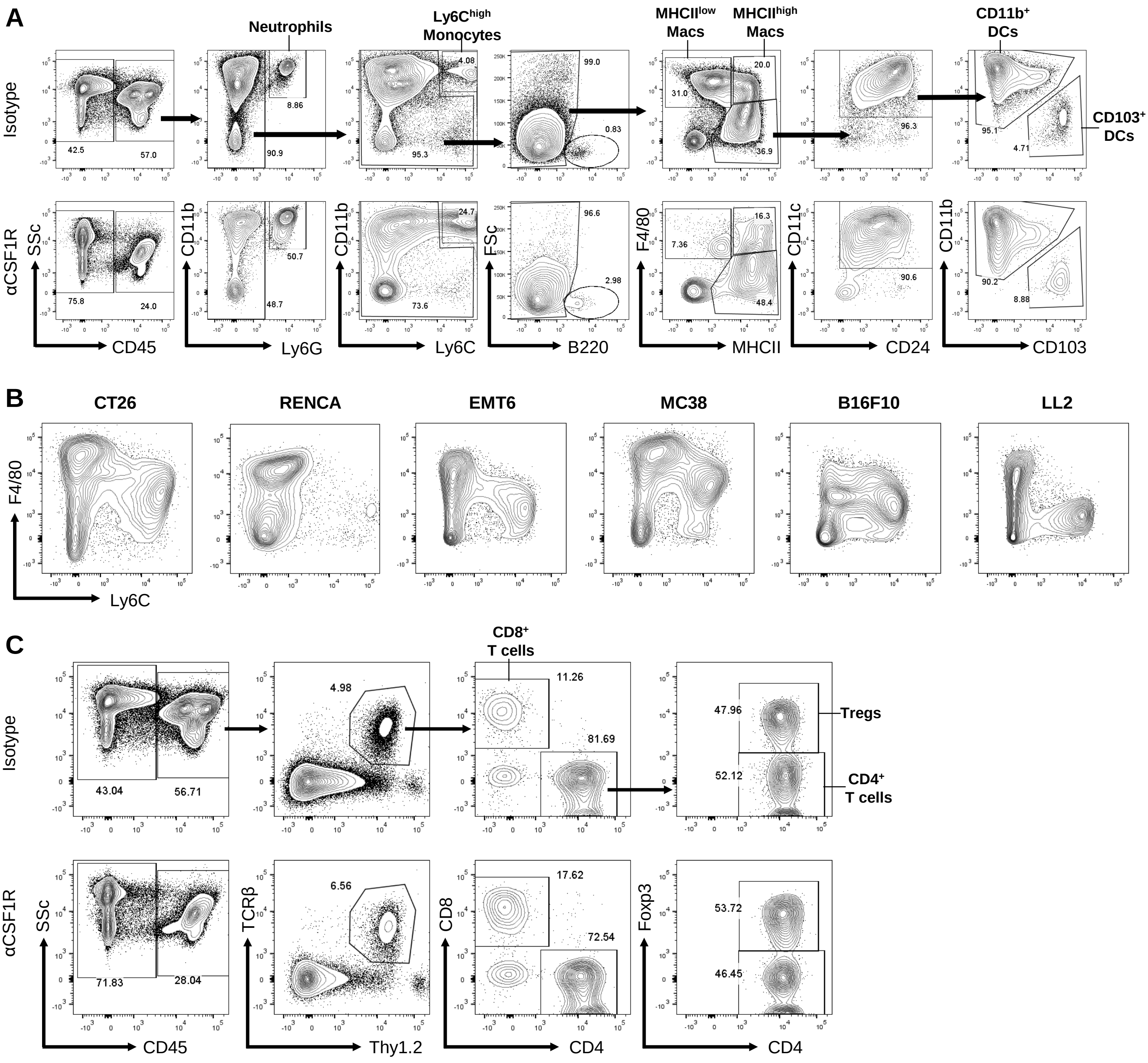

Supplemental Figure 1. Tumor immune cell gating strategy.

a Myeloid cell gating strategy used to identify neutrophils, Ly6C^{high} classical monocytes, MHCII^{high} and MHCII^{low} macrophages, and CD103^{+} (cDC1) and CD11b^{+} (cDC2) dendritic cells. Note, this gating scheme does not allow identification of tumor-associated Ly6C^{low} non-classical monocytes, which may be carried over into the macrophage gates. **b** F4/80 verse Ly6C staining on cells from CD45^{+}Ly6G^{-} (non-neutrophil) gate shows the ability to distinguish F4/80^{high} macrophages from F4/80^{int}Ly6c^{high} classical monocytes using this gating scheme. **c** T cell gating strategy. Histograms in (**a**) and (**c**) are from RENCA tumors, with the top row showing a representative isotype control-treated tumor and the bottom row showing a representative αCSF1R-treated tumor. Cell populations of interest are indicated.

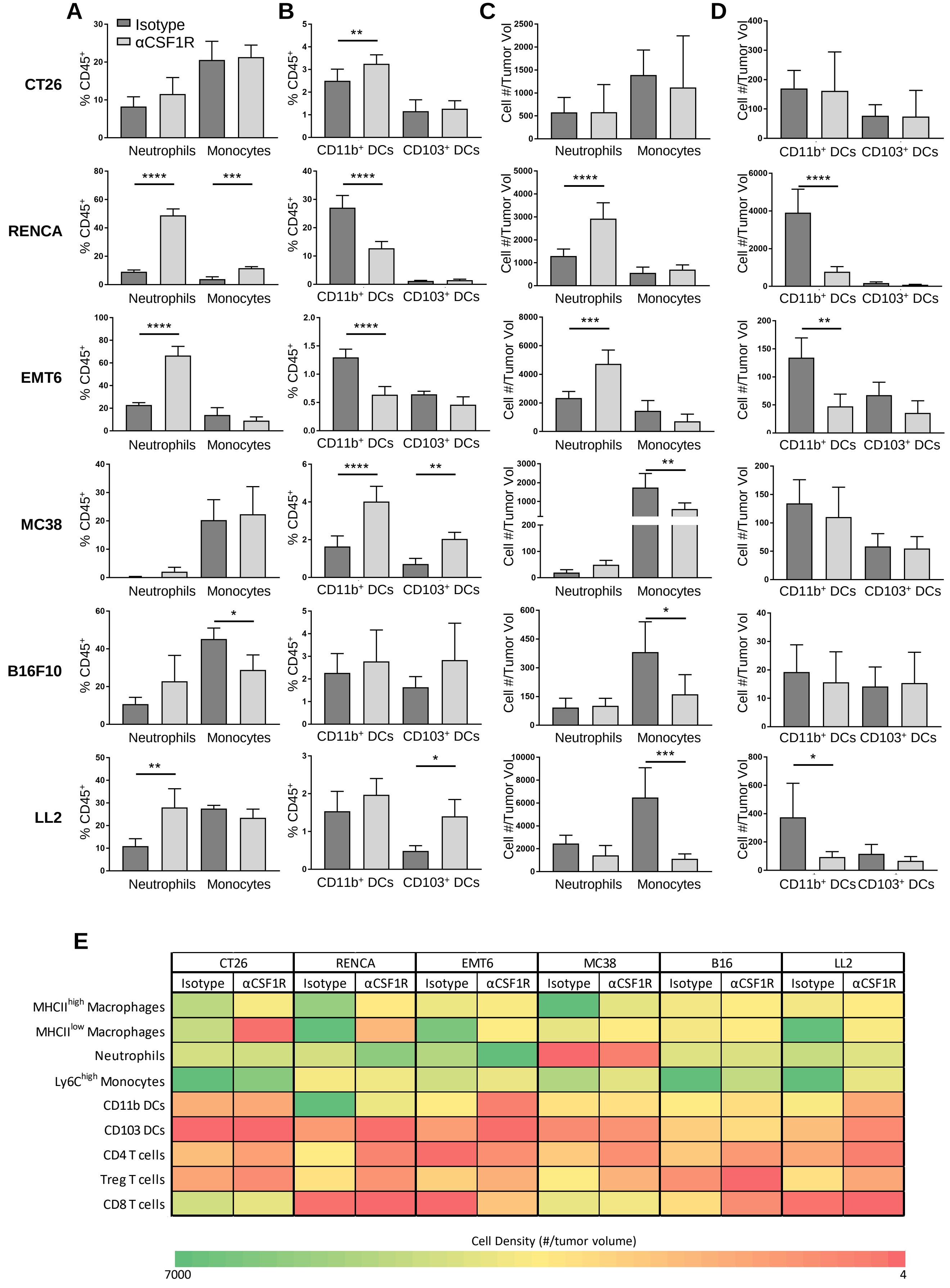
A
B
C
D
Isotype
αCSF1R
CT26
RENCA
EMT6
MC38
B16F10
LL2
% CD45+
Cell #/Tumor Vol
Neutrophils
Monocytes
CD11b+ DCs
CD103+ DCs
E
CT26
RENCA
EMT6
MC38
B16
LL2
Isotype
αCSF1R
MHCII^high Macrophages
MHCII^low Macrophages
Neutrophils
Ly6C^high Monocytes
CD11b DCs
CD103 DCs
CD4 T cells
Treg T cells
CD8 T cells
Cell Density (#/tumor volume)
7000
4

Supplemental Figure 2. Changes in innate immune populations after αCSF1R treatment.

100mm^3 subcutaneous tumor-bearing mice were treated with 4 doses of αCSF1R or isotype control antibody and tumor-associated immune populations were assessed by flow cytometry as in Supplemental Figure 1. $CD11b^+Ly6G^+$ neutrophils and $CD11b^+Ly6C^+$ monocytes (**a, c)** and $CD24^+CD11c^+$ DCs subdivided based on their expression of CD103 or CD11b (**b, d)** are shown as a percentage of total $CD45^+$ immune cells (**a, b)** or cell density (cell number/tumor volume) (**c, d);** n=5-10 animals/group. **e** Heatmap representing cell density (cell number/tumor volume) of specific immune populations within different tumor models following treatment with αCSF1R or isotype control antibodies, as indicated. One-way ANOVA, Tukey's multiple comparisons. *p<0.05, **p<0.01, ***p<0.001, ****p<0.0001.

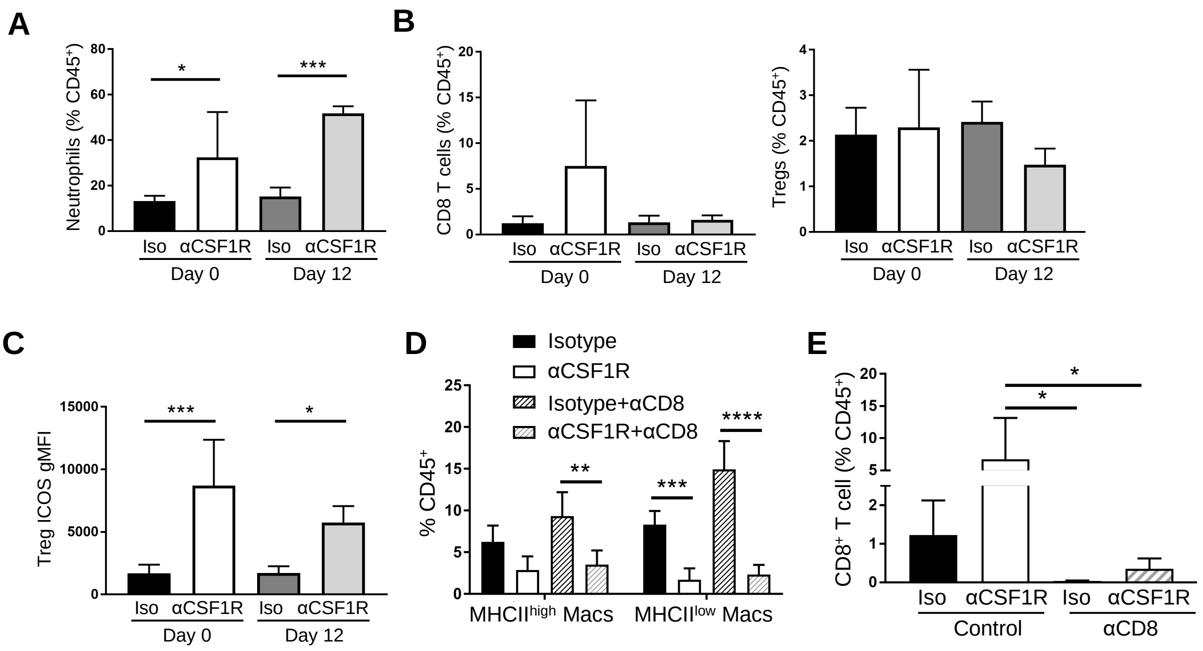

Supplemental Figure 3. Early vs late initiation of αCSF1R therapy in Renca tumors.

a-c Flow cytometry of RENCA tumors treated on day 0 or day 12 with αCSF1R or control antibodies. Neutrophils (**a**) and CD8 and Treg cells (**b**) shown as a percentage of $CD45^+$ immune cells. **c** Geometric mean fluorescence intensity (gMFI) for ICOS staining on Treg populations. **d, e** Depletion of TAMs (**d**) and $CD8^+$ T cells (**e**) in RENCA tumors isolated from mice treated at day 0 with αCSF1R, αCD8, or control antibodies as indicated in Fig. 3d; n=5-7 animals/group. One-way ANOVA, Tukey's multiple comparisons. *p<0.05, **p<0.01, ***p<0.001, ****p<0.0001.

	Day 0						Day 12					
	Isotype		Anti-CSF1R				Isotype		Anti-CSF1R			
	-dCT	St Dev	-dCT	St Dev	Fold Change	P Value	-dCT	St Dev	-dCT	St Dev	Fold Change	P Value
**BATF3**	-4.78	0.46	-5.40	1.43	-1.54	0.3088	-5.13	0.74	-6.80	0.78	-3.1661	0.0090
**CD11b**	0.87	0.27	-1.59	0.86	-5.51	<0.00001	0.76	0.09	-4.17	0.39	-30.409	<0.00001
**CD11C**	-1.55	0.11	-2.51	0.38	-1.95	0.0001	-1.45	0.16	-3.39	0.34	-3.8455	<0.00001
**CD3E**	-3.65	1.12	-2.26	0.75	2.61	0.0300	-4.18	0.70	-5.43	0.77	-2.3868	0.0358
**CD4**	-3.04	0.49	-4.13	0.57	-2.12	0.0362	-3.06	0.49	-6.42	1.06	-10.2652	<0.00001
**CD45**	1.00	0.17	-0.40	0.57	-2.64	<0.00001	0.62	0.29	-2.24	0.11	-7.2457	<0.00001
**CD8A**	-5.14	1.77	-2.47	0.95	6.35	0.0053	-5.28	0.89	-5.73	0.84	-1.3743	0.5802
**CMPK2**	-1.87	0.55	-0.57	0.66	2.48	0.0004	-2.06	0.13	-2.21	0.25	-1.1102	0.5898
**CSF1R**	2.13	0.10	-1.18	0.68	-9.93	<0.00001	2.00	0.21	-2.45	0.23	-21.9692	<0.00001
**CXCL10**	-1.81	0.66	-0.24	1.14	2.97	0.0030	-2.06	0.19	-3.78	0.44	-3.291	0.0009
**CXCL9**	-2.69	1.55	0.69	1.29	10.44	0.0006	-3.21	0.73	-3.49	0.97	-1.208	0.7171
**Eselectin**	-9.18	0.36	-6.90	1.54	4.84	0.0298	-8.30	1.62	-7.84	0.29	1.3822	0.5854
**F4/80**	-0.75	0.22	-3.83	1.13	-8.42	<0.00001	-1.12	0.40	-6.05	0.46	-30.5187	<0.00001
**GZMB**	-7.41	1.94	-3.50	1.59	15.04	0.0026	-8.17	1.28	-9.06	0.66	-1.8423	0.4683
**IFIT1**	-3.36	0.35	-1.64	0.62	3.28	0.0001	-3.68	0.46	-3.65	0.46	1.0199	0.9252
**IFNG**	-8.12	0.89	-4.91	1.67	9.27	0.0095	-9.28	1.10	-10.04	1.06	-1.6972	0.5091
**IL6**	-8.15	0.96	-6.13	1.52	4.08	0.0345	-7.79	0.90	-7.70	0.21	1.0669	0.9051
**IL7**	-5.61	1.00	-3.98	0.53	3.11	0.0023	-5.13	0.60	-4.88	0.28	1.1867	0.5650
**NKp46**	-7.85	2.06	-5.33	0.85	5.75	0.0157	-7.87	1.06	-8.05	0.77	-1.1335	0.8579
**PRF1**	-7.86	1.04	-4.31	0.69	11.70	0.0018	-9.37	1.71	-9.22	0.61	1.1115	0.8646
**Pselectin**	-6.07	1.36	-3.98	0.57	4.27	0.0013	-6.09	0.40	-5.36	0.33	1.6649	0.1636
**T-bet**	-7.67	1.72	-5.85	1.06	3.54	0.0869	-8.12	2.14	-8.45	0.61	-1.2577	0.7715
**TIGIT**	-6.24	0.79	-4.90	0.53	2.54	0.0109	-6.41	0.53	-6.95	0.82	-1.4585	0.2309
**XCL1**	-7.64	1.55	-5.33	1.39	4.96	0.0107	-8.19	0.93	-8.37	0.34	-1.13	0.8257

Supplemental Figure 4. Early initiation of αCSF1R therapy potentiates inflammatory gene expression in RENCA model.

Fluidigm qRT-PCR data from Figure 4. -dCT values were calculated by normalizing expression of individual genes to the *Ipo8*, *Tbp*, and *Hrpt* housekeeping genes. Average -dCT values and standard deviation are shown; n=4-5 animals/group. Fold change and p-value (two-tailed t-test) for αCSF1R vs isotype control comparisons were calculated using the General Linear Model function of Array Studio software. Fold change is on a linear scale; values ≥1 represents higher gene expression in αCSF1R group relative to isotype group while fold change ≤-1 represents lower gene expression in αCSF1R group relative to isotype group.

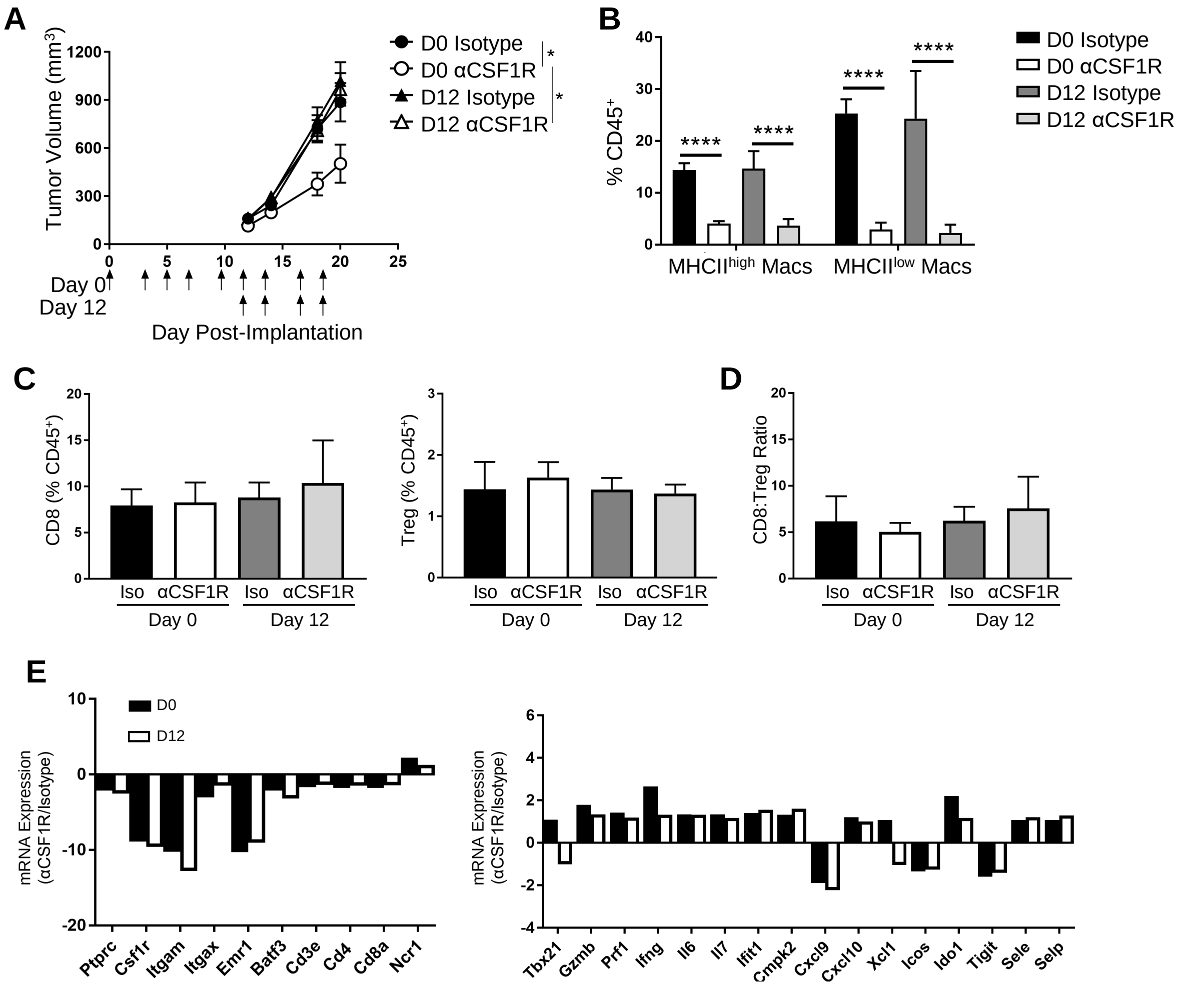

Supplemental Figure 5. Effect of early vs late dosing of αCSF1R in CT26 tumors.

a Tumor growth curves for mice treated as indicated by arrows starting on day 0 or 12 post-tumor implantation; n=10 animals/group. One-way ANOVA, Sidak's multiple comparisons (αCSF1R vs isotype control at D0 and D12, αCSF1R D0 vs αCSF1R D12). **b-d** Frequency of TAMs (**b**) and $CD8^+$ T cells and Treg cells (**c**) as a percentage of $CD45^+$ cells and ratio of $CD8^+$ T cells to Tregs (**d**) in tumor as measured by flow cytometry; n=5 animals/group. One-way ANOVA, Tukey's multiple comparisons. **e** Fluidigm qRT-PCR analysis. Graphs represent average -dCT values for αCSF1R over isotype-treated animals; n=4-5 animals/group. *p<0.05, ***p<0.001, ****p<0.0001
